# MRA Toolbox v. 1.0: a web-based toolbox for predicting mixture toxicity of chemical substances in chemical products

**DOI:** 10.1038/s41598-022-13028-0

**Published:** 2022-05-25

**Authors:** Jongwoon Kim, Myungwon Seo, Jiwon Choi, Minju Na

**Affiliations:** Chemical Safety Research Center, Korea Research Institute of Chemical Technology (KRICT), Daejeon, 34114 Republic of Korea

**Keywords:** Environmental sciences, Software, Chemical safety, Cheminformatics, Green chemistry

## Abstract

The chemical risk assessment paradigm is shifting from “substance-based” to “product/mixture-based” and from “animal testing” to “alternative testing” under chemical regulations. Organisms and the environment may be exposed to mixtures rather than a single substance. Conducting toxicity tests for all possible combinations is impractical due to the enormous combinatorial complexity. This study highlights the development and application case studies of Mixture Risk Assessment Toolbox, a novel web-based platform that supports mixture risk assessment through the use of different prediction models and public databases. This integrated framework provides new functional values for assessors to easily screen and compare the toxicity of mixture products using different computational techniques and find strategic solutions to reduce the mixture toxicity in the product development process. The toolbox (https://www.mratoolbox.org) includes four additive toxicity models: two conventional (Concentration Addition; and Independent Action) and two advanced (Generalized Concentration Addition; and Quantitative Structure–Activity Relationship-based Two-Stage Prediction) models. We demonstrated the multiple functions of the toolbox using three cases: (i) how it can be used to calculate the mixture toxicity, (ii) those for which safety data sheet (SDS) only indicating representative toxicity values (EC_50_; and LC_50_), and (iii) those comprising chemicals with low toxic effects.

## Introduction

Progress in the chemical industry over the past several decades has culminated in the mass production of a wide diversity of products. In Europe, over 100,000 chemicals have been manufactured and marketed, and 200–300 new chemicals are evaluated annually^1^. In response to global chemical regulations, the paradigm of chemical risk assessment is shifting from ‘substance-based’ to ‘product/mixture based and from ‘animal testing’ to ‘alternative testing’. Living organisms and the environment are exposed to mixtures rather than single substances. Humans are exposed to about 100 different chemicals daily. These substances are found in consumer products and foods^2^. Even at their no observed effect concentrations (NOEC), certain chemicals may contribute to mixture toxicity because of the cocktail effect^3^. European chemical regulations regarding mixtures may be substance- and product-based such as Registration, Evaluation, Authorization, and Restriction of Chemicals (REACH); Biocidal Products (BPR); Food and Feed Additives (FFA); Placing of Plant Protection, Products (PPP); and Classification, Labelling, and Packaging of Substances and Mixtures (CLP). They may also be media- and process-based such as Integrated Pollution and Prevention Control (IPPC) and the Water Framework Directive (WFD). REACH defines mixtures as compositions of at least two different substances that are not chemically bonded. Several previous studies revealed that mixture toxicity may be caused by additive or synergistic effects among chemicals^3–9^. The combined effects of a chemical mixture may be classified as additivity, synergism (*i.e*., more than additivity), and antagonism (*i.e*., less than additivity)^10^. Mixture toxicity tests cannot be performed on every possible component combination because of the enormous combinatorial complexity involved. For example, twenty chemicals may be combined into 190 binary combinations and over one million mixtures of three or more compounds^8,11^. In addition, the in-depth mechanism of mixture toxicity are complex, and using conventional experimental tests, it is significantly expensive and time consuming to clearly understand the effect of the combination and composition of chemical mixtures, target species, and environmental stressors on the mixture toxicity^12^. Recently, various computational toxicology approaches based on machine learning and deep learning techniques have been developed and applied to predict the toxicity of chemicals and mixtures^13–16^. The advantage of computational toxicology is that it provides the opportunity to rapidly consider large datasets and effectively calculate the toxicity of chemicals, even at low levels of exposure and across species^17^. Thus, computational toxicology methods are expected to become the core technologies for conducting mixture risk assessment. Mixture toxicity prediction was traditionally based on additive toxicity caused by the sum of effects concentration or the responses of chemicals in the mixture^18^. Conventionally, the concentration addition (CA)^19^ and independent action (IA, also known as the response addition)^20^ models have been frequently used to estimate the additive toxicity of substances with the same modes of action (MoAs) and those with different MoAs, respectively. The CA model has been the default for mixture risk assessments under the pertinent chemical regulations because it is easy to use. In the CA model, the half-maximal effective concentration (EC_50_) of single substances may be used to calculate those of mixtures. The CA model usually generates more conservative prediction values than the IA model. The latter cannot determine the sum of the responses (%) of mixture components below their NOEC. Cedergreen et al.^21^ reviewed the accuracy of the CA and IA models at estimating the toxicity of binary mixtures. Their study had 159 data points for 98 mixtures with different MoAs. About 20% and 10% of these mixtures were correctly calculated by the CA and IA models, respectively. About half the data points were improperly predicted by both models. MoAs data are required for all mixture components to establish which model is suitable for the analysis. Because of their underlying assumptions, both models consider either similar or dissimilar MoAs. Efforts have been made to avoid the conceptual limitations of the conventional CA and IA models. The Generalized Concentration Addition (GCA) model was developed to predict additive toxicity for chemical substances with low toxicity effects^22^. The quantitative structure–activity relationship-based two-stage prediction (QSAR-TSP) model was developed to predict the toxicity of mixture components with different MoAs. To this end, a chemical clustering method is applied based on machine learning techniques, structural similarities among the substances to estimate MOAs of components. Mixture toxicity is predicted by integrating both CA and IA concepts^23^.

Although advanced prediction models based on different machine learning algorithms have been developed, one of the main challenges in applying them to mixture risk assessment is the lack of accessible tools for those who are not proficient at working with programming languages. There have been very few available web tools for mixture risk assessments. The Chemical Mixture Calculator^24^ (http://www.chemicalmixturecalculator.dk) and the Monte Carlo Risk Assessment Toolbox^25^ (MCRA, https://mcra.rivm.nl) are probabilistic models that assess the risks of combined dietary and non-dietary exposures to various chemicals. These tools have mainly focused on cumulative exposure assessments to evaluate risk characterization. They compare exposure and hazard threshold levels of target mixtures under the assumption of dose-additivity in mixture risk assessment rather than mixture toxicity assessment of cocktail effects. They require numerous calculations and detailed hazard and exposure data for mixture components. More practical, user-friendly, web-based, regulatory-compliant tools may be useful for the chemical industry; these tools will facilitate the accessibility of the mixture risk assessment so that the industry can consider and screen potential cocktail effects in chemical mixtures during product development. In this aspect, this study highlights the development and application of case studies using Mixture Risk Assessment (MRA) Toolbox, a novel web-based platform for supporting mixture risk assessment, including different prediction models and public databases. This integrated framework of the toolbox was designed to provide new functional values for assessors to easily screen and compare the toxicity of mixture products using different computational techniques, and determine strategic solutions to reduce the mixture toxicity in the product development process.

Therefore, in this study, MRA Toolbox v. 1.0 was developed by integrating various predictive models with related databases on the web platform for computational mixture risk assessment. It rapidly considers and compares the estimated cocktail effects of chemical mixtures using different underlying concepts frequently employed in this field. MRA Toolbox is freely available at https://www.mratoolbox.org, and pursues the safe and sustainable-by-design framework of chemical products; it provides a chemical safety technology platform for virtually testing the toxicity of chemical mixture products by changing the mixture formulations and finding alternatives to hazardous. The present study thus exhibits the current version of MRA Toolbox that predicts the combined toxicity of mixture components. It uses conventional CA and IA models as lower-tier approaches and the more advanced additive toxicity models GCA and QSAR-TSP as higher-tier approaches. The mixture toxicity values are based on available user input data and predicted and automatically compared by all four models in the toolbox. Moreover, chemical properties search by interfacing with the chemical name, CAS number, molecular weight, molecular structure of PubChem DB (https://pubchem.ncbi.nlm.nih.gov) will^26^ be provided. Dual data saving modes are furnished to ensure user data confidentiality and security. The multiple functions of MRA Toolbox were demonstrated using three different case studies: (i) how it can be used to calculate the toxicity of mixtures, (ii) those for which SDS only indicate EC_50_/LC_50_, and (iii) those comprising chemicals with low toxic effect^27–29^.

## Methods

### System architecture of MRA Toolbox v. 1.0

The web application design and implementation of MRA Toolbox requires (i) system software (e.g., operating system, web development kit, and database management system), (ii) various computational models (e.g., mathematical, and statistical algorithms), (iii) scientific software (e.g., molecular modeling software, programming languages and libraries for machine learning), and (iv) open data sources.

**Figure 1:**
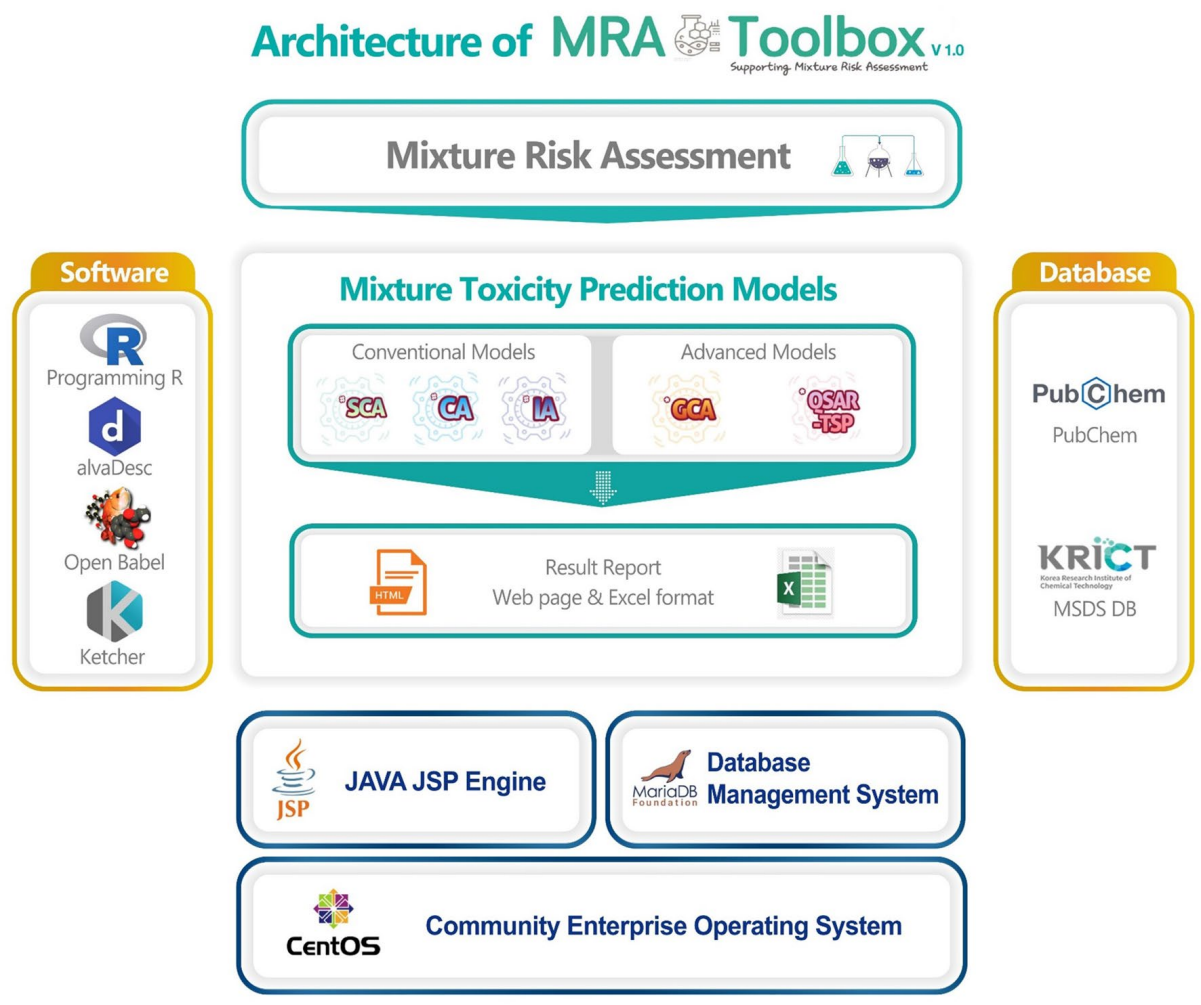
Architecture of MRA Toolbox v. 1.0.

Figure 1 illustrates the system architecture of MRA Toolbox. System software programs employed in the toolbox were the Community Enterprise Operating System (CentOS)^30^ v. 8.3, Java Server Pages^31^ for the web development environment of graphical user interfaces (GUIs), and MariaDB v. 10.3.27 (MariaDB Foundation, https://mariadb.org/) for the database management system. As computation models for estimating mixture toxicity, four predictive models were implemented in the toolbox: two conventional models (e.g., CA and IA) as lower-tier models, and two advanced models (e.g., GCA and QSAR-TSP) as higher-tier models described in the introduction. The predictive model algorithms were implemented via the programming language R v. 4.0.2^32^ in the toolbox wherein the CA, IA and GCA were implemented using the modified ‘mixtox’ package v. 1.3.2 in R^33^. We partly modified the original mixtox package to be fitted for the toolbox purposes to cover a wider range of model input data with additional fitting curve equations, unit conversion functions, and automatic determination of predictable DRC ranges as follows:

(i) Ten dose–response curve (DRC) equations were added to the mixtox package; in total, 17 DRC regression models were set up in the toolbox (Supplementary Table S1);
(ii) Unit conversion features were additionally implemented to enable the inter-conversion of nM, μM, mM, ug/L, and mg/L; and
(iii) Automatic determination of predictable DRC ranges was added as an essential function for the toolbox. This was done as the full DRC of a mixture could not be calculated at a certain higher concentration in cases where there was a toxic effect of mixture components. This additional function enables the toolbox to estimate the mixture toxicity within the available effect range of low toxic components by automatically selecting a concentration section wherein it could be calculated.

The algorithm of the QSAR-TSP model, assuming that similar structures have similar MoAs, was implemented in R. In QSAR-TSP, MoAs of components in the product are divided into groups based on their structural similarity calculated by descriptors. When grouping of components for MoA, the calculated descriptors were compressed into fewer principal components that address the total data while minimizing information loss by the principal component analysis (PCA)^34^ in R. PCA results were grouped based on the *k*-means clustering method and calculated sequentially by CA and IA models. In cases where mixture products include only two components (*i.e.,* binary mixtures), their MoAs were predicted according to structural features calculated to extended connectivity fingerprint (ECFP) level 6 based on the RxnSim package v. 1.0.3 in R^35^ since the QSAR-TSP and the *k*-means clustering method are applicable to more than three mixture components. For binary mixtures, the structural similarity for binary mixtures is thus calculated by using the Tanimoto coefficient as an indicator based on the ECFP level 6 results^36^. If the structural similarity between two components is more than 0.7, both chemicals are assumed to have a similar MoA and the CA model is employed to calculate their mixture toxicity. In the opposite case, the IA model is applied.

As scientific software programs for the toolbox, two molecular modeling software programs were used for calculating chemical structural information: alvaDesc software v. 2.0.0^37^ and Open Babel software v.3.3.1^38^. alvaDesc is a very powerful commercial software which calculates more than 5000 molecular descriptors representing the physicochemical properties of a chemical structure. In cooperation with an alvaDesc development team, the software was successfully embedded in the toolbox with the following limitation: users cannot directly access results calculated from alvaDesc since its calculation process, based on the user’s input data, is only calculated from the back-end server computer. Open Babel software widely used as an open source was applied in optimizing chemical structures in the toolbox. For users to directly draw and upload their molecular structures into the tool, Ketcher^39^, as on open software, was also embedded for the molecular modeling process.

As open data sources, MRA Toolbox interfaces with the PubChem DB (NIH, USA) and includes the material safety data sheet (MSDS) DB of the Chemical Integrated Management System (KRICT CMS System) of the Korea Research Institute of Chemical Technology [http://krict-cms.krict.re.kr (available in Korean language mode)]. The information on the main hazard classification (GHS, Globally Harmonized System) and risk management measures are provided from the MSDS DB. Chemical information, including molecular structure, CAS number, molecular weight, physicochemical properties, etc., are provided to users by connecting to the PubChem DB.

### Work process of MRA Toolbox v. 1.0

**Figure 2:**
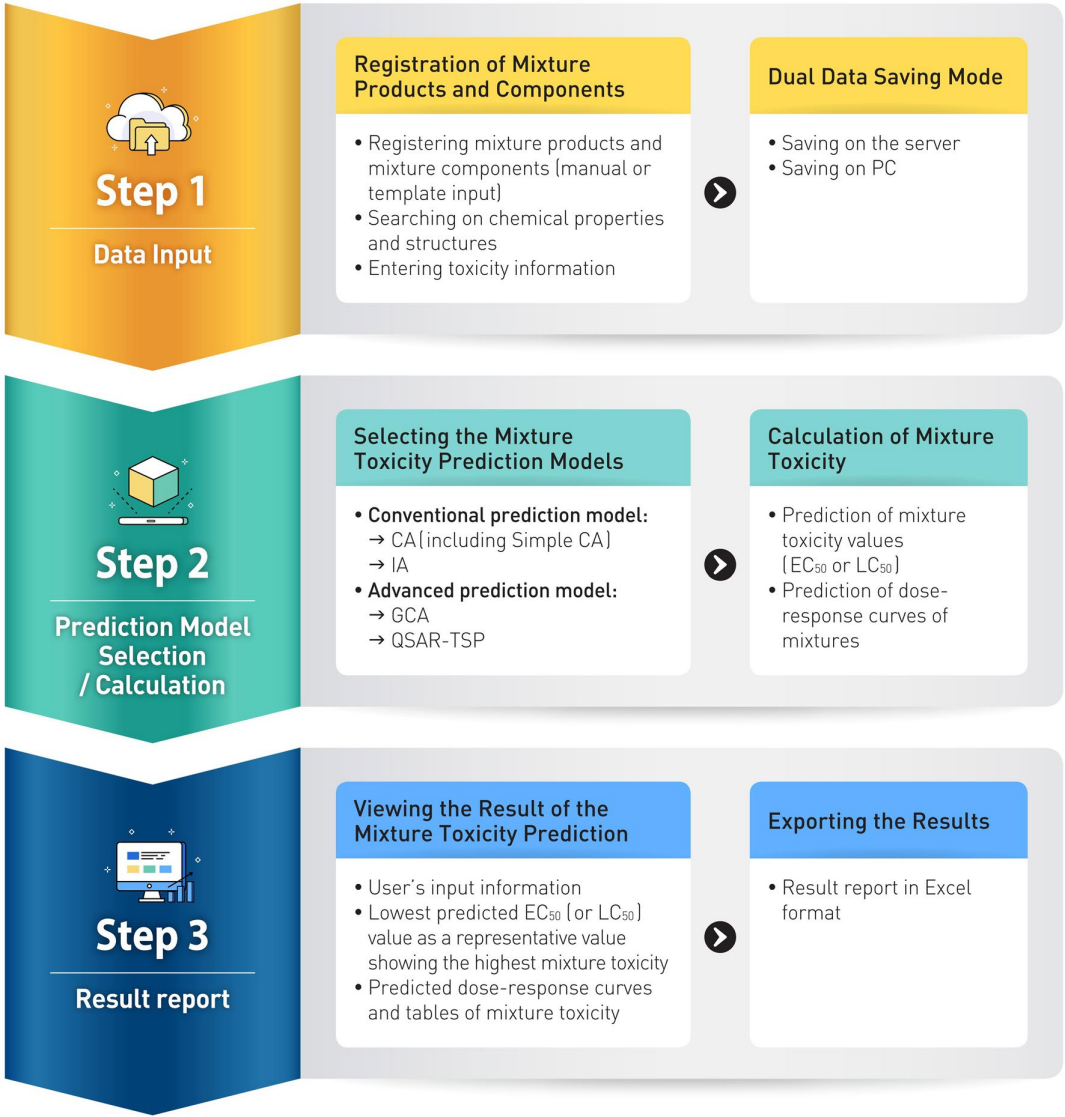
Overview of MRA Toolbox v. 1.0.

MRA Toolbox was designed to have a step-by-step work process as shown in Fig. 2:

(i) [Step I for data input] In step I, for mixture risk assessment, a user registers mixture product information such as product name and description in the registration area. Data related to mixture components (chemical name, CAS number, composition, toxicity information, etc.) are entered by manually inputting or uploading the template. User searches chemical properties and downloads structures from the PubChem DB based on the chemical name or CAS number. If a chemical is not found, the user can directly upload it in MoL or SDF file format or draw and enter a chemical structure in MRA Toolbox using the Ketcher. Classification and risk management measures can be searched for in the MSDS DB associated with a chemical name or CAS number. Toxicological information such as regression models and model parameters are required for predicting the mixture toxicity with different prediction models (CA, IA, GCA, and QSAR-TSP);
(ii) [Step II for predictive model selection/calculation] In step II, predictive models are selected to estimate the mixture toxicity of the chemical product. According to the user data input level, a user selects the mixture toxicity prediction model. If the MoA information has the same or different mixture components, the user can use conventional models. Only the Simple CA model estimating mixture EC_50_ can be applied if toxicity information is not entered. The user can employ the GCA model in case mixture components include low toxicity effects. The QSAR-TSP model can be used for complex mixtures with both similar and dissimilar MoA. Calculation of mixture toxicity provides mixture toxicity values such as EC_50_ or LC_50_ and DRCs of mixtures; and
iii) [Step III for Result reporting] In step III, a user can confirm the result of the mixture toxicity prediction. The result consists of the lowest predicted EC_50_ value of the mixture, predicted DRCs and tables of the mixture, and the user’s input information. The predicted mixture toxicity is reported on the website, and users can download this information in Excel format.

### Dual data saving modes for data confidentiality

We designed dual data saving modes for the information security of users in the MRA Toolbox as follows: (i) saving on PC only, and (ii) saving on the server. The saving on PC only mode allows users to enter and save the input data temporarily during the web browser session. The saving on server mode allows the users’ input data to be conserved on the MRA Toolbox server.

### Data collections for case studies of MRA Toolbox applications

To demonstrate multiple functions of the MRA Toolbox, we collected three datasets for the case studies (e.g., a complex mixture; a mixture including only EC_50_ or LC_50_ and a mixture containing chemicals with low toxic effect) in literature as follows:

(i) [Dataset 1] a 23-component mixture^27^ as a representative complex mixture was selected for Case Study 1 with considering more than ten components having their detail information on compositions, DRC equations, and modes of action (Supplementary Table S2);
(ii) [Dataset 2] a SDS of a coating product, a primer for adhesives, having four main components^28^ was collected for Case Study 2 (Table 2) in which the composition, EC_50_s and LC_50_s of mixture components were described, but additional information on their DRC equations was not included;
(iii) [Dataset 3] a mixture^29^ consisting of three perfluorinated carboxylic acids, partial agonist on peroxisome proliferator-activated receptor alpha activity, was selected for Case Study 3 for considering low efficacy compared with positive control (Supplementary Table S3).

The dataset 3 of mixtures having low toxic components does not provide the value of parameters for regression models for DRC, but a DRC graph only. In this case, the data points of DRC were manually extracted from the graph with WebPlotDigitizer^40^ v 4.4. Thereafter, the parameters of DRC were calculated by fitting according to a Hill three-regression model using SigmaPlot v. 14. 0 (Systat Software Inc., San Jose, CA, USA). SigmaPlot software was used for in-detail plotting of the results of Case Study 3. WebPlotDigitizer and SigmaPlot are not included in the toolbox.

We selected the QSAR-TSP model to predict the mixture toxicity of complex mixtures with both similar and dissimilar MoA. The GCA model was selected to predict mixture toxicity, including components of low toxic effects. CA and IA models were selected to compare the predicted results with the QSAR-TSP and GCA models. We selected a simple CA model to estimate the mixture toxicity in general toxicity data, including EC_50_ or LC_50_ only.

## Results

We developed the web-based MRA Toolbox for supporting mixture risk assessment and pursuing the safe and sustainable-by-design framework of chemical products. It uses various computational techniques to screen the potential cocktail effects of chemical mixtures. It can be used to find methods of reducing the mixture toxicity in the product development process. We designed a simple and user-friendly GUI based on the system architecture to operate the toolbox following the work process. Moreover, we implemented dual data saving modes for data confidentiality. The multiple functions of the toolbox were demonstrated through three case studies: (i) toxicity prediction of a complex mixture consisting of various MoAs chemicals, (ii) prediction of mixture toxicity using only EC_50_ or LC_50_ in a SDS, and (iii) mixture toxicity prediction including chemicals with low toxic effect.

## Development of the MRA Toolbox v. 1.0

### Data input and graphical user interface

**Figure 3:**
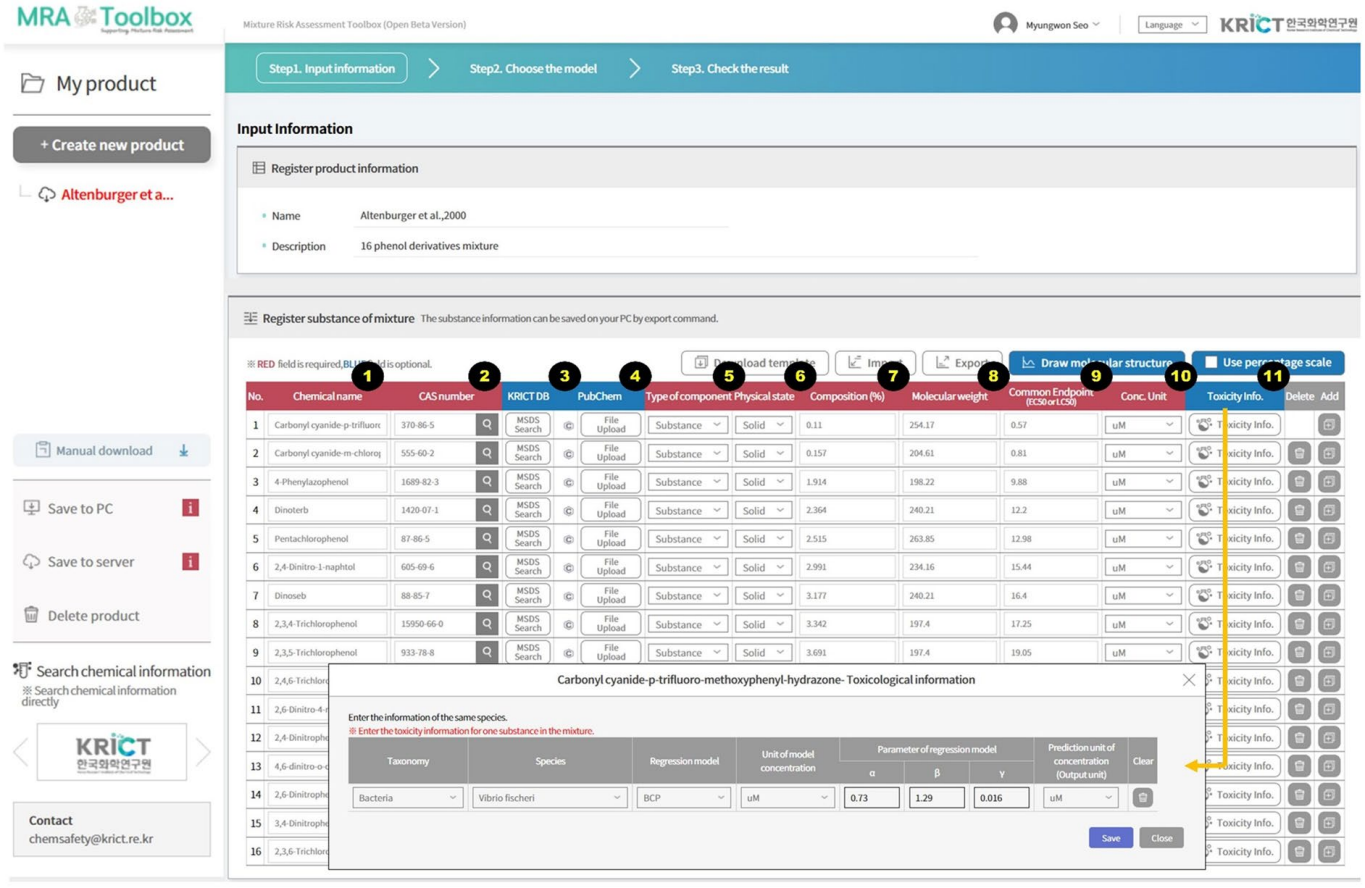
MRA Toolbox graphical user interface (GUI) to enter chemical product, composition, and hazard data.

The MRA Toolbox has a simple GUI to enter data for chemical mixtures and their components. Figure 3 shows the product information registration area in Step I. There, the input data can be categorized as mandatory or optional. The mandatory and optional data fields (cells) are in red and blue, respectively. The main data fields consist of (i) chemical name; (ii) CAS number; (iii) KRICT DB; (iv) PubChem DB; (v) component type such as substance or sub-mixture; (vi) physical state; (vii) composition (%); (viii) molecular weight; (ix) common endpoint such as EC_50_ or LC_50_; (x) concentration unit; and (xi) toxicity information (Fig. 3).

Chemicals containing the letters being entered in the GUI are listed via the auto-complete feature and the PubChem DB interface. Hence, CAS numbers are automatically retrieved and may be utilized to search for the target chemical. For chemicals not automatically detected, it can manually enter the CAS number. Classification and risk management measures described in the MSDS and associated with a chemical name or CAS number may appear in the MSDS search if the CAS number or chemical name is present in the KRICT DB.

Chemical structures of input data are automatically downloaded if a chemical structure corresponding to a CAS number or chemical name is included in the PubChem DB. If the molecular structure cannot be found in the PubChem DB, or the structure is new without a CAS number, it can draw structure using the Ketcher, a drawing tool embedded in the toolbox. Also, it can directly upload a structure as a MOL or SDF file format.

The component type and physical state are designed dropdown menu. The substance or sub-mixture can be selected in the component type, solid, liquid, or gas can be chosen in the physical state. Mixture composition (%), molecular weight, common endpoints, and concentration units can be entered via the GUI. In the data field, the common endpoint is the value of EC_50_ or the median lethal concentration (LC_50_). The concentration unit is designed with a dropdown menu containing five units. It must be entered along with the toxicity endpoint value. The molecular weight of a chemical substance is automatically retrieved via the PubChem DB interface when the CAS number is entered.

The toxicity information includes DRCs such as regression models, model concentration unit, regression model parameters such as α, β, and γ, and predicted concentration unit for calculating outputs in a common unit. Though the toxicity information is optional, the toxicity information is nonetheless required for predicting the DRCs of the mixture toxicity of targets with different prediction models (CA, IA, GCA, and QSAR-TSP). All the input data can be entered into the toolbox manually as well as it allows mass data input features using an excel template for the user convenience. MRA Toolbox GUI provides both English and Korean languages.

### Predictive model selection and calculation

**Figure 4:**
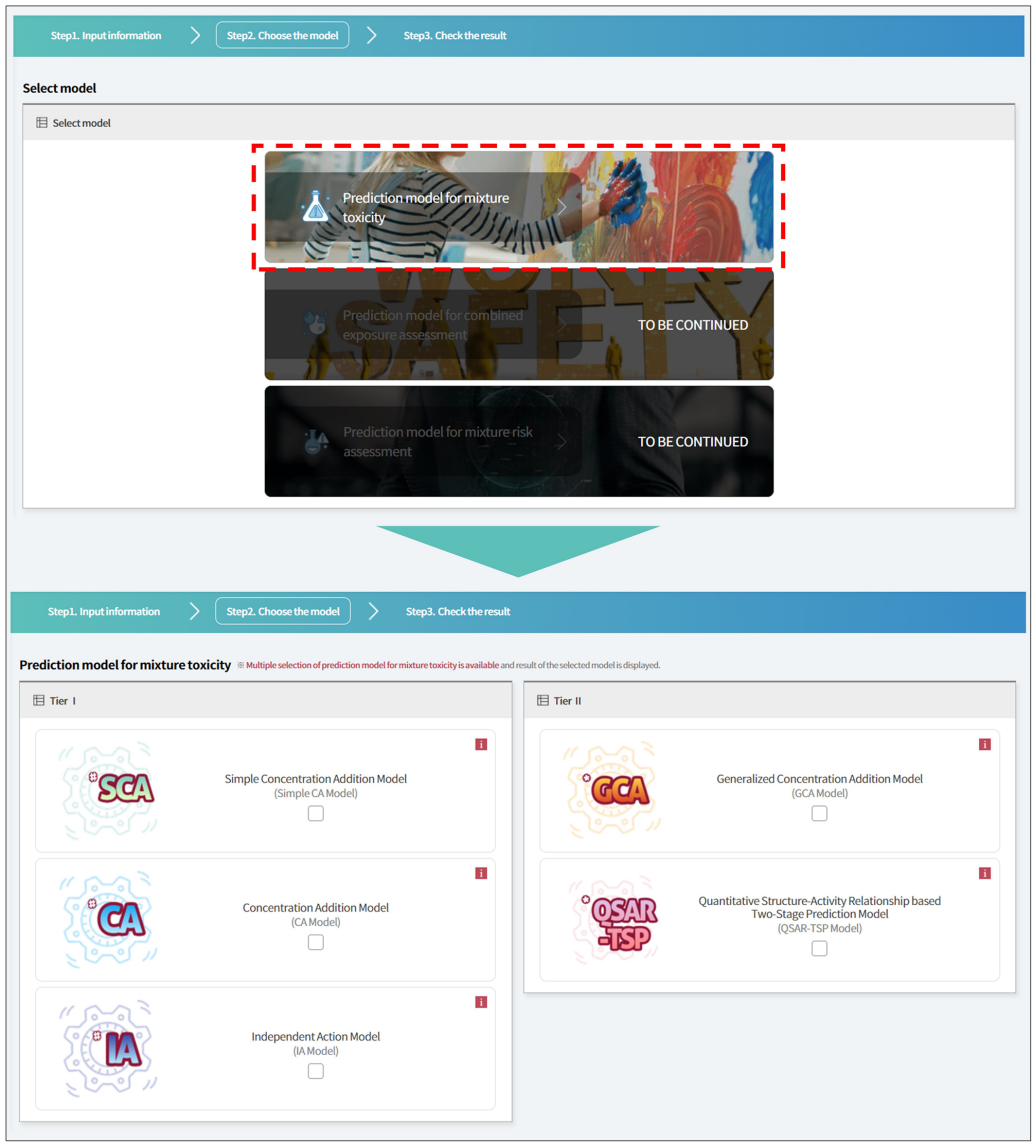
MRA Toolbox graphical user interface (GUI) to select mixture toxicity predictive models.

The MRA Toolbox ultimately pursues estimating combined risk for various chemical mixtures using the mixture toxicity prediction, exposure assessment, and mixture risk assessment modules. The toolbox currently focuses on implementing the mixture toxicity prediction module. The other two modules will be integrated into later versions of MRA Toolbox. We implemented a model selection page as a checkbox to carry out more than two model’s calculations. The user’s selection enables the prediction of mixture toxicity by using between one model and a maximum of five models (Fig. 4).

### Result report and graphical output

**Figure 5:**
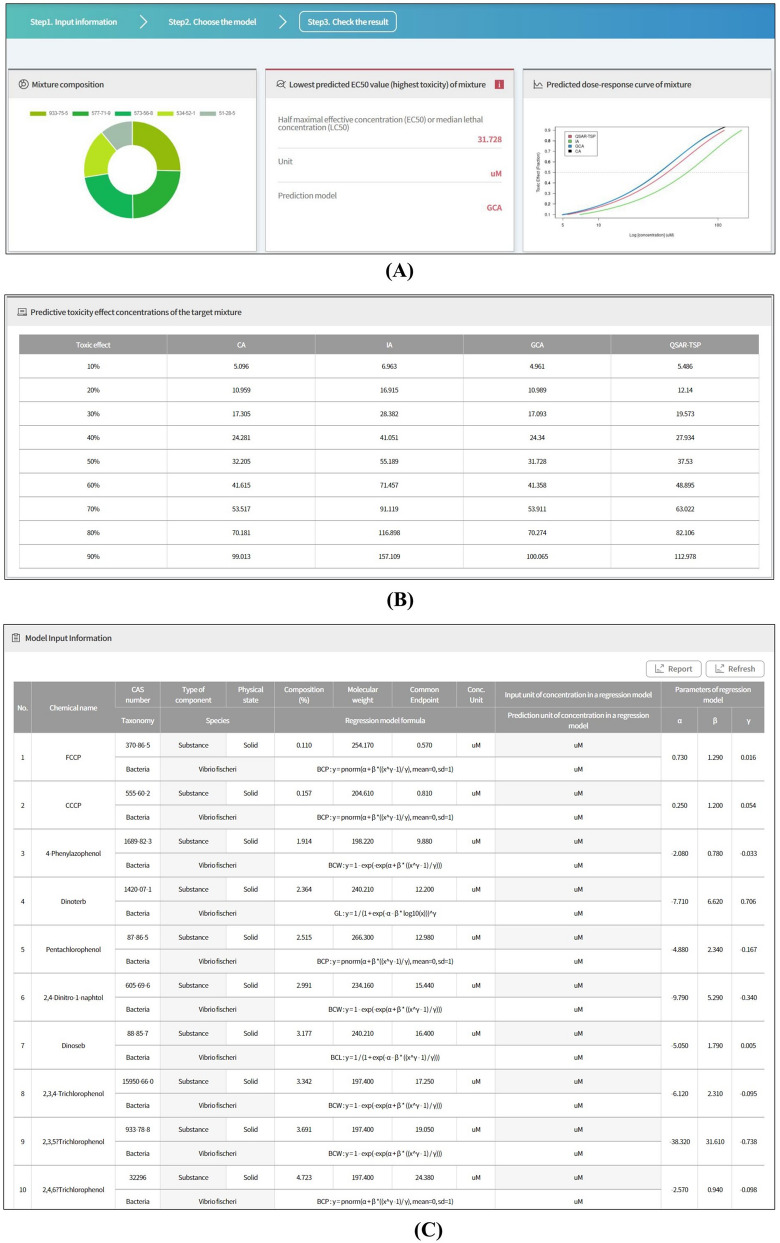
MRA Toolbox GUI for reporting prediction results. (**A**) Graphical information for mixture composition; representative EC_50_ as lowest value and DRCs of target mixture predicted by selected models; (**B**) toxicity effects (%) vs. effective concentrations computed by models; and (**C**) user input data for all mixture components.

The MRA Toolbox provides summary information including mixture composition, lowest predicted EC_50_ value, predicted DRCs, and tabulated predicted mixture toxicity effects and input data (Fig. 5). Mixture composition is displayed in the form of a donut graph wherein the top five chemical substances are illustrated along with their CAS numbers. The lowest predicted EC_50_ or LC_50_ values for mixtures reflect the highest toxicity values forecasted by the selected model(s). Predicted mixture DRCs are calculated by user-selected models and plotted as sigmoid graphs. If only the simple CA model is selected, either EC_50_ or LC_50_ is provided and no DRC is plotted for the mixture (Fig. 5A). The predicted mixture toxicity effects values are tabulated (Fig. 5B). Here, the mixture toxicity values predicted by each model show in the range of 10–90%. Mixture component input data are also tabulated (Fig. 5C).

### Implementation of dual data saving modes

MRA Toolbox provides dual data saving modes for data confidentiality. In saving on PC only, the information entered is temporarily saved and is only available during the web browser session. If the web browser session is logged off, information cannot be the after logging back on. The mode of saving on the server is information to be conserved on the MRA Toolbox server. After the user exits the system, the information will still be visible upon re-entry.

## Result of application case studies

### Case Study 1: Toxicity prediction for a complex mixture consisting of substances with both similar and dissimilar modes of action

**Figure 6:**
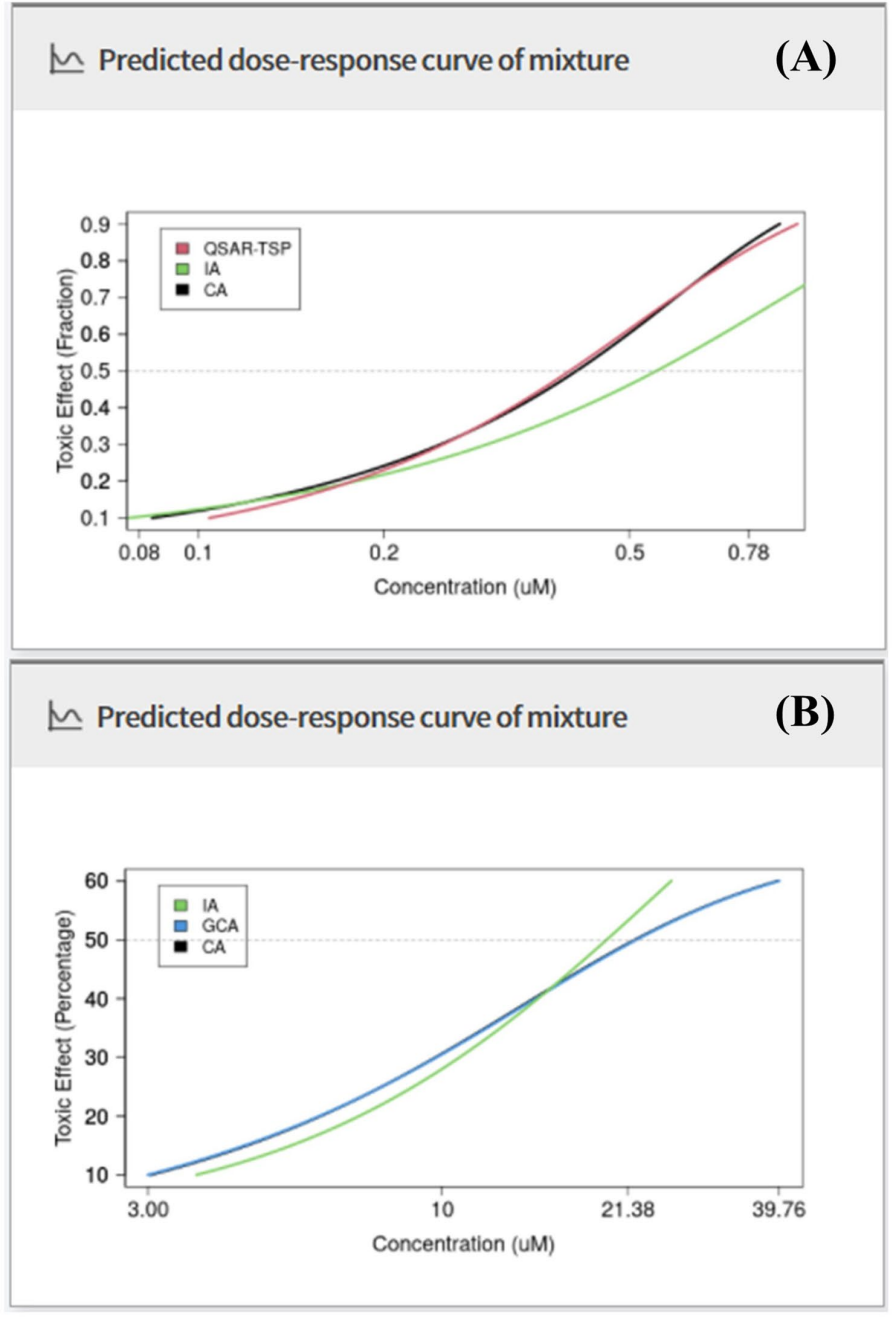
Comparisons of CA, IA, GCA, and QSAR-TSP predictions. (**A**) Case Study 1; and (**B**) Case Study 3.

Complex mixtures may comprise various chemicals with both similar and dissimilar MoAs. Conventional CA and IA models theoretically require that the MoAs of all chemicals in the mixture are specified. The CA and IA make binary assumptions that the MoAs of all chemicals in a mixture is either similar or dissimilar, respectively. Hence, both models can be limited to predicting the mixture toxicity of complex mixtures composed of similar and dissimilar MoAs chemicals, basically. MRA Toolbox includes the QSAR-TSP model as an integrated addition model that can consider such complex mixtures with combining the CA and IA concepts. In Case Study 1, MRA Toolbox was used to demonstrate how the toxicity of a complex mixture could be calculated via the CA, IA, and QSAR-TSP models and using data on DRCs of mixture components from a previously published study^27^ (Supplementary Table S2). This study examined the effects of a mixture of 23 pesticides with different MoA groups on the reproduction of the green alga *Scenedesmus vacuolatus* strain 211-15^27^. The QSAR-TSP model performed the best^23^. MRA Toolbox plotted DRCs and predictive toxicity effect concentrations for the target mixture in graph and table form, respectively (Fig. 6A; Table 1). For the predicted DRCs, users may compare toxic effects in the range of 10–90%. For the predicted DRCs, users may apply the QSAR-TSP model to predict mixture toxicity with a higher degree of accuracy. The QSAR-TSP model is optimized to predict the toxicity of mixtures comprising at least three components with similar or dissimilar MoAs. For binary mixtures, the QSAR-TSP model assumes their MoAs by calculating their structural similarity (for detailed information of the structural similarity calculation, refer to the Methods section and Kim et al.^23^). On this basis, the mixture toxicity is then predicted by the CA or IA model.

**Table 1:** Summary of predicted effective concentrations of mixture for Case Study 1.

Toxic effect (%)	Predicted effective concentrations of mixture (μM) [CI]		
	CA	IA	QSAR-TSP
10	0.083 [0.057; 0.144]	0.077 [0.038; 0.160]	0.106 [0.078; 0.175]
20	0.166 [0.132; 0.193]	0.180 [0.135; 0.225]	0.180 [0.141; 0.212]
30	0.246 [0.208; 0.258]	0.293 [0.242; 0.314]	0.251 [0.208; 0.265]
40	0.327 [0.287; 0.339]	0.416 [0.359; 0.431]	0.324 [0.279; 0.336]
50	0.409 [0.373; 0.430]	0.552 [0.496; 0.578]	0.403 [0.360; 0.425]
60	0.497 [0.472; 0.529]	0.707 [0.662; 0.751]	0.491 [0.458; 0.527]
70	0.594 [0.583; 0.636]	0.890 [0.867; 0.955]	0.596 [0.579; 0.646]
80	0.711 [0.698; 0.758]	1.125 [1.112; 1.201]	0.732 [0.720; 0.788]
90	0.876 [0.809; 0.904]	1.478 [1.373; 1.528]	0.943 [0.864; 0.981]

### Case Study 2: Toxicity prediction for a complex mixture based on safety data sheet (SDS) information

General toxicity data for mixture components can be provided in SDS. However, DRCs and certain other toxicity data are not specifically included in the SDS. For components omitted from the DRCs, the DRC of mixture toxicity cannot be estimated using CA, IA, GCA, and QSAR-TSP. Nevertheless, MRA Toolbox provides a simple CA model that predicts mixture toxicity even when DRCs are unavailable for certain chemical components. Half-maximal effective concentrations (EC_50_) and half-maximal lethal concentrations (LC_50_) for mixtures may be predicted by the simple CA model when EC_50s_ or LC_50s_ of the mixture components are known for the components. For the MRA Toolbox case study, a coating product consisting of four components served as the mixture^28^. The mixture toxicity of the coating product was predicted based on the simple CA model and using its composition ranges and ecotoxicity data (*e.g*., EC_50_ for *Daphnia magna*) publically described in SDS (Table 2). In the worst-case scenario, maximum composition values of mixture components were selected to estimate the EC_50_ of their mixture using the simple CA model. As MRA Toolbox furnishes a simple CA model, users may still estimate mixture toxicity even when DRCs of components are missing.

**Table 2:** Composition and toxicity data of mixture components in SDS, and EC_50_ of mixture predicted by the simple CA model for Case Study 2.

Substance	CAS RN	MW (g/mol)	Composition range (%)	EC_50_ (mg/L-48 h, D. magna)
Toluene	108-88-3	92.140	70–**75**	11.500
n-Butanol	71-36-3	74.120	1–**5**	1983
2-Butoxyethanol	111-76-2	118.170	1–**5**	1000
Ethyl alcohol	64-17-5	46.070	1–**5**	9268
Mixture	–	–	73–90	15.314

### Case Study 3: Toxicity prediction of a mixture containing low-toxicity chemicals

Original programming codes for the CA, IA, and GCA calculations in the ‘mixtox’ R package included in MRA Toolbox basically generate mixture toxicity values for toxic effects in the range of 10–90%. Thus, the CA and IA algorithms in the original ‘mixtox’ package can display error message (*e.g.*, not available) in the calculation when some chemicals may have low toxic effect ranges that maximal toxic effect is lower than 50%. All model algorithms were coded in MRA Toolbox to solve this limitation by automatically selecting a concentration section which could be calculated for low toxic effect chemicals. In Case Study 3 having low-toxicity chemicals, MRA Toolbox was used to predict mixture toxicity via the CA, IA, and GCA models using data from an earlier case study on low toxic effects (Supplementary Table S3). That study predicted the effects of activating peroxisome proliferation activated receptor alpha (PPARα) with mixtures of three perfluorinated carboxylic acids and three sulfonic acids, respectively^29^. MRA Toolbox plotted DRCs for mixture toxicity and toxic effect concentrations in the range of 10–60% based on the predictive models (Fig. 6B; Table 3). Case Study 3 shows the toxicity of mixtures including low-toxicity chemicals can be appropriately estimated and compared by the CA, IA, and GCA models in MRA Toolbox within the available DRC ranges of mixture components.

**Table 3:** Summary of predicted effective concentrations of mixture for Case Study 3.

Toxic effect (%)	Predicted effective concentrations of mixture (μM) [CI]		
	CA	IA	GCA
10	3.031 [− 3.700; 12.397]	3.660 [2.209; 5.538]	3.001 [− 3.024; 11.626]
20	6.084 [0.303; 10.812]	7.112 [5.965; 7.775]	6.033 [0.321; 10.706]
30	9.736 [3.565; 13.236]	10.759 [9.393; 11.743]	9.836 [3.722; 13.112]
40	14.582 [6.361; 21.749]	14.825 [13.706; 16.167]	14.611 [7.533; 20.860]
50	22.030 [16.745; 30.916]	19.599 [18.970; 20.840]	22.216 [17.722; 30.432]
60	39.549 [29.062; 48.642]	25.566 [23.671; 27.071]	39.756 [29.935; 47.993]

## Discussion

Toxicity experiments have been conducted using in vitro and animal models to understand the combined toxic effects of individual chemicals in mixtures. However, the number of possible combinations is virtually extremely large. Hence, not every mixture can be evaluated by these approaches. Non-testing methods have been widely applied in the attempt to overcome experimental limitations and predict chemical and mixture toxicity. In the non-testing methods, the chemical mixture calculator and the MCRA Toolbox were developed for mixture risk assessment^24,25^. The chemical mixture calculator is a web-based tool that considers similar chemical classes and exposure routes in specific target organs and performs mixture risk assessments based on dose-additive effects. The MCRA toolbox considers combined exposure of multiple chemicals and assesses the risks of mixtures based on probabilistic methods. Since both tools mainly focus on conducting the cumulative exposure assessment in mixture risk assessment, they may have a limitation to predict cocktail effects using predictive models such as CA and IA. However, the MRA Toolbox regards the cocktail effect of the chemical mixture and uses it to estimate mixture toxicity.

In the aspect of application, the MRA Toolbox provides benefits that risk assessors can apply various methods, covering different situations shown in the case studies, to the prediction of mixture toxicity easily, and promptly compare their results in mixture risk assessment. The toolbox has the conventional CA and IA models which have been frequently considered under chemical regulations. In addition, it also contains the QSAR-TSP and GCA models as advanced methods for calculating additive toxicity in cases where targets are complex mixtures including both similarly and dissimilarly acting compounds, or they are combinations of components with low toxic effects which may be critical limitations of the CA and IA models basically. In the aspect of the usability, the MRA Toolbox as a web-based platform provides a convenient user interface for predicting mixture toxicity, and offers systematic outputs in a report form including summary figures and tables. The toolbox provides prediction results such as DRCs in the overall effect range of 10–90% when appropriate input data sets are provided. It displays them in table and graph formats rather than single data points such as EC_50_.

Nevertheless, MRA Toolbox v.1.0 has several limitations. First, the user must enter DRC model parameters of all mixture components to obtain a DRC of their mixture toxicity using different models. If such data is insufficient, the predictive models in the toolbox may not effectively predict mixture toxicity for entire effective ranges. Second, only aquatic species as target organisms can be selected for estimating mixture toxicity in the toolbox. Toxicity data on aquatic species are allowed to be input in the predictive models. To expand the target species list, more lists of taxonomic groups should be added to the species database of the toolbox. Third, the ultimate goal of the MRA Toolbox should be prediction of risks which can be occurred by different chemical combinations. Mixture risk assessment requires not only the toxicity of mixture components but also their human and environmental exposures. However, the current version of MRA Toolbox focuses mainly on the toxicity of chemical mixtures. Thus, MRA Toolbox must be expanded by integrating exposure assessment models and risk characterization methods for combined exposures to chemical mixtures.

## Conclusion and outlook

In the present study, we developed and introduced the web-based MRA Toolbox v. 1.0 supporting the mixture risk assessment. The freely available MRA Toolbox assesses mixture toxicity based on different prediction models (*e.g.,* CA, IA, GCA, and QSAR-TSP) and includes a user-friendly GUI. We demonstrated the multiple functions of the toolbox and showed how it can be used to calculate the toxicity of complex mixtures, those for which SDS only indicate EC_50_/LC_50_, and those comprising chemicals with low toxic effect. For further studies to expand the applicability and increase the user-friendliness of the toolbox, it will be upgraded by adding other prediction models and linking it to available databases and toolkits providing experimental and predicted data, *e.g.,* European Chemicals Agency (ECHA) DB (https://echa.europa.eu) and Organisation for Economic Co-operation and Development (OECD) QSAR Toolbox Web API (https://qsartoolbox.org), to fill in data gaps users have. This is to overcome the application limit of the toolbox mainly caused by lack of data. In addition, the risk characterization methods for combined exposures will be added to the toolbox for assessors to finally carry out the risk assessment of mixture products. The list of taxonomic groups in the toolbox will be also updated to consider more toxicity data on different species.

In the future, MRA Toolbox will pursue a safe and sustainable-by-design framework of chemical products to find a strategic solution to effectively reduce the risk of mixture toxicity of chemicals by changing mixture composition or substituting components with alternatives. It can contribute to public health and sustainable chemistry as a chemical safety technology platform. A variety of case studies are necessary to support mixture risk assessment continuously and maintain the reliability of the toolbox.

## Supplementary Information

Supplementary Information.

## Data Availability

MRA Toolbox v. 1.0 is freely available at https://www.mratoolbox.org. All data generated or analyzed in this study is publicly available and is included in this article (and its supplementary information files).

## Abbreviations

BPR: Biocidal products
CA: Concentration addition
CI: Confidence interval
CLP: Classification, labelling, and packing of substances and mixtures
DRC: Dose–response curve
ECFP: Extended connectivity fingerprint
ECHA: European chemicals agency
EC_50_: Half-maximal effective concentration
FFA: Food and feed additives
GCA: Generalized concentration addition
GHS: Globally harmonized system
GUI: Graphical user interface
IA: Independent action
IPPC: Integrated pollution and prevention control
LC_50_: Median lethal concentration
MCRA: Monte Carlo risk assessment toolbox
MoA: Mode of action
MRA: Mixture risk assessment
MSDS: Material safety data sheet
NOEC: No observed effect concentration
OECD: Organisation for Economic Co-operation and Development
PCA: Principal component analysis
PPP: Placing of plant protection, products
PPARα: Peroxisome proliferation activated receptor alpha
QSAR-TSP: Quantitative structure–activity relationship-based two-stage prediction
REACH: Registration, Evaluation, Authorization, and Restriction of Chemicals
SDS: Safety data sheet
WFD: Water Framework Directive
