# Supplementary material for: MRA Toolbox v. 1.0: a web-based toolbox for predicting mixture toxicity of chemical substances in chemical products
Source: Sci Rep. 2022 May 25;12:8880. doi: 10.1038/s41598-022-13028-0 (PMC9132927; doi:10.1038/s41598-022-13028-0)
Supplement: Supplementary file 1 — Supplementary Information. [file 41598_2022_13028_MOESM1_ESM.pdf]

Supplementary Information

MRA Toolbox® v. 1.0: A web-based toolbox for predicting mixture toxicity of chemical substances in chemical products

Jongwoon Kim[§,*], Myungwon Seo[§], Jiwon Choi, Minju Na

Chemical Safety Research Center, Korea Research Institute of Chemical Technology, Daejeon, 34114, Republic of Korea
[§]These authors contributed equally: Jongwoon Kim and Myungwon Seo.

[*]jkim@krict.re.kr

Supplementary Table S1. Regression models used for calculating concentration-response curve to MRA Toolbox® v. 1.0

No.	Regression model	Function
1	Hill	$E(c)=\frac{1}{1+\left(\frac{\alpha}{c}\right)^{\beta}}$
2	Hill two	$E(c)=\frac{\beta c}{\alpha+c}$
3	Hill three	$E(c)=\frac{\alpha}{1+\left(\frac{\gamma}{c}\right)^{\beta}}$
4	Weibull	$E(c) = 1 - \exp(-\exp(\alpha + \beta\log_{10}(c))$
5	Weibull three	$E(c) = \gamma(1 - \exp(-\exp(\alpha + \beta\log_{10}(c))))$
6	Logit	$E(c) = \frac{1}{1 + \exp(-\alpha - \beta\log_{10}(c))}$
7	Logit three	$E(c) = \frac{\gamma}{1 + \exp(-\alpha - \beta\log_{10}(c))}$
8	Box-Cox-Weibull (BCW)	$E(c) = 1 - \exp\left(-\exp\left(\alpha + \beta\left(\frac{c^{\gamma} - 1}{\gamma}\right)\right)\right)$
9	Box-Cox-Logit (BCL)	$E(c) = \frac{1}{1 + \exp(-\alpha - \beta\frac{c^{\gamma} - 1}{\gamma})}$
10	Generalized Logit (GL)	$E(c) = \frac{1}{[1 + \exp(-\alpha - \beta\log_{10}(c)]^{\gamma}}$
11	Probit	$E(c) = \frac{1}{2\pi}\int_{-\infty}^{\alpha+\beta\log_{10}(c)} \exp\left(\frac{-u^2}{2}\right) du = \Phi(\alpha + \beta\log_{10}(c)$
12	Box-Cox-Probit (BCP)	$E(c) = \Phi(\alpha + \beta\frac{c^{\gamma}-1}{\gamma})$
13	Sigmoid	$E(c) = \frac{\alpha}{1 + \exp\left(-\frac{c - \gamma}{\beta}\right)}$
14	Logistic	$E(c) = \frac{\alpha}{1 + \left(\frac{c}{\gamma}\right)^{\beta}}$
15	Chapman	$E(c) = \alpha(1 - \exp(-\beta c)^{\gamma}$
16	Gompertz	$E(c) = \alpha\cdot \exp(-\exp\left(\frac{-c - \gamma}{\beta}\right)$
17	Weibull drc	$E(c) = 1 - \exp(-\exp(\alpha\log(c) - \log(\beta)))$

E(c): the fractional effect at concentration c ($0 \leq E \leq 1$); c: concentration; α, β, and γ: model parameters (corresponding statistical estimates); Φ: cumulative normal (Gaussian) distribution; exp(x) = e^x.

Supplementary Table S2. Physical properties and parameters for regression models for concentration-response curves of *Scenedesmus vacuolatus* strain 211-15 (Junghans et al. 2006)

Substance	CAS RN	MW [g/mol]	RM [a]	Parameter			EC_{50} [μM]
				α [b]	β [c]	γ [d]	
Single component							
2,4-D	94-75-7	221.04	GL	-37.540	11.106	0.1392	856
Aclonifen	74070-46-5	264.67	BCW	2.402	0.408	-0.3400	0.0297
Alachlor	15972-60-8	269.77	W	4.009	5.127		0.140
Atrazine	1912-24-9	215.69	GL	6.765	17.391	0.1118	0.180
Bromoxynil	1689-84-5	276.91	L	-19.600	9.267		130
Carbofuran	1563-66-2	221.26	W	-4.564	1.978		132
Chloridazon	1698-60-8	221.65	W	-2.375	2.777		5.29
Cycloxydim	101205-02-1	325.47	W	-5.232	1.990		278
Ethofumesate	26225-79-6	286.35	W	-2.126	1.108		38.7
Ioxynil	1689-83-4	370.92	W	-3.785	2.229		34.2
Isofenphos	25311-71-1	345.39	GL	-3.373	2.186	0.4219	7.76
Isoproturon	34123-59-6	206.29	BCW	1.246	1.073	-0.0235	0.228
Isoxaflutole	141112-29-0	359.32	W	-5.313	2.529		90.4
Lenacil	2164-08-1	234.30	GL	14.991	14.338	0.1845	0.0495
Linuron	330-55-2	249.10	W	1.769	2.020		0.0877
MCPA	94-74-6	200.62	P	-4.501	1.551		798
Metamitron	41394-05-2	202.22	W	-0.995	1.912		2.13
Metolachlor	51218-45-2	283.80	BCW	0.239	3.156	0.4930	0.818
Pendimethaling	40487-42-1	281.31	W	5.752	2.957		0.00853
Terbuthylazine	5915-41-3	229.71	W	4.165	3.908		0.0693
Thifensulfuron-methyl	79277-27-3	387.38	L	-2.093	1.837		13.8
Triasulfuron	82097-50-5	401.82	W	0.093	1.684		0.533
Tribenuron-methyl	101200-48-0	395.39	W	0.670	1.735		0.253
Mixture							
Mixture of 23 substances	-	-	BCW	1.090	1.896	0.3659	0.406

[a] Regression models; [b] Height; [c] Slope; and [d] Center point

Supplementary Table S3. Physical properties and parameters for regression models for concentration-response curves of PPARα ligands (Nielsen et al. 2022)

**Substance**	**CAS RN**	**MW [g/mol]**	**RM** [a]	**Parameter**			**EC_{50} [μM]**
				α [b]	β [c]	γ [d]	
Single component							
Perfluoroheptanoic acid (PFHpA)	375-85-9	364.06	Hill	111.300	1.003	43.760	43.760
Perfluorooctanoic acid (PFOA)	335-67-1	414.07	Hill	83.203	1.241	8.147	9.667
Perfluorononanoic acid (PFNA)	375-95-1	464.08	Hill	60.800	1.756	4.497	7.783
Mixture							
Equipotent mixture	-	-	Hill	80.350	1.709	12.150	11.617

[a] Regression models; [b] Height; [c] Slope; and [d] Center point
